# Patient characteristics of completion and dropout of mentalization-based treatment for adolescents with conduct disorder

**DOI:** 10.3389/fpsyg.2024.1390169

**Published:** 2024-10-02

**Authors:** Sophie Hauschild, Drago Dragovic, Lea Kasper, Esther Sobanski, Svenja Taubner

**Affiliations:** ^1^Institute for Psychosocial Prevention, University Hospital Heidelberg, Heidelberg, Germany; ^2^Psychological Institute, Heidelberg University, Heidelberg, Germany; ^3^Department of Child and Adolescent Psychiatry, Lucerne, Switzerland; ^4^Medical Faculty of Mannheim, Department of Psychiatry and Psychotherapy, Central Institute of Mental Health, Heidelberg University, Heidelberg, Germany; ^5^Deutsches Zentrum für Psychische Gesundheit (German Center for Mental Health), Heidelberg, Germany

**Keywords:** conduct disorder, adolescence, personality pathology, cluster analysis, mentalization-based treatment, dropout, stepwise logistic regression

## Abstract

**Introduction:**

Conduct disorder (CD) is a severe mental disorder in youth. Yet, providing psychological interventions for adolescents with CD is challenging. This patient group is often characterized by risk factors for therapy dropout such as, e.g., CD symptoms and being in middle adolescence. On the other hand, little is known about characteristics of adolescents with CD who complete treatment. To gain more insight into what might become a successful therapy with adolescents with CD, this study explores baseline characteristics and drop-out occurrence in patients with CD referred to mentalization-based treatment for adolescents with CD (MBT-CD). More specifically, this study aims at identifying clusters of adolescent patients based on age, CD symptom severity and personality pathology at the beginning of treatment which may have come along with a higher or lower dropout occurrence.

**Methods:**

Following implications of an elbow plot, a 3-means cluster-analysis was conducted on self-report baseline data of *N* = 32 adolescents with CD (*n* = 11 dropouts, *n* = 21 completers) who participated in a feasibility study on MBT-CD. Additionally, in an exploratory stepwise logistic regression, variables were explored as potential predictors of dropout.

**Results:**

Cluster 1 consisted of *n* = 14 adolescents, of whom *n* = 8 (57%) dropped out. Cluster 2 consisted of *n* = 5 adolescents of whom 1 (20%) dropped out. Cluster 3 consisted of *N* = 13 adolescents, of whom only *n* = 2 (15%) dropped out. Cluster 2 showed descriptively the highest CD symptom severity. While adolescents in Clusters 1 and 3 showed in part similarities in CD symptom severity, personality pathology was descriptively markedly higher in Cluster 1. In the stepwise logistic regression, only intimacy personality pathology was identified as potential predictor for dropout.

**Discussion:**

This study’s exploratory findings point to different types of adolescents with CD coming along with different chances for therapists to conduct a (successful) psychotherapy. Herein, low personality functioning in the intimacy domain, rather than CD symptoms as aggressiveness, may play a crucial role. While findings are limited by the small sample size, they may be able to shed increasing light on conducting (successful) psychotherapy with a scarcely researched patient group.

## Introduction

Conduct disorder is a frequent, severe and cost-intensive mental disorder in children and adolescents (Polanczyk et al., 2015; Rivenbark et al., 2018). Conduct disorder is characterized by repeated aggressive behavior against humans or animals, destruction of property or severe rule-breaking. Due to the overlap between mental health and judicial relevance of CD symptom behavior, programs aiming to reduce symptom behavior vary and may consist of (court-ordered) rehabilitation, incarceration and/or psychotherapeutic interventions. Amongst different approaches, psychological interventions were identified as the most effective in reducing symptom behavior (e.g., Bakker et al., 2017), while judicial processing increases symptom behavior (Petrosino et al., 2010). However, providing psychological interventions for individuals with CD is challenging, and it seems even more so for adolescents as compared to children. For adolescents with mental disorder symptoms in general, dropout rates of outpatient psychotherapy are high (de Haan et al., 2013; O’Keeffe et al., 2018; Jørgensen et al., 2021). In addition, CD symptom behavior and being in middle adolescence specifically was found to increase the likelihood of dropping out of therapy (Baruch et al., 1998; O’Keeffe et al., 2018). Moreover, most adolescents with a CD diagnosis do not initiate therapy themselves (cmp. Koelch et al., 2019; Hauschild et al., 2023). Rather, therapy sessions may be court-ordered or demanded by other third parties, e.g., by the school to avoid expulsion. Being sent to treatment in this manner bears the risk of further minimizing adolescents` autonomy, which is a salient need in this developmental phase. This adds to the challenge of treating individuals with CD, as not being self-referred impedes treatment success and has been identified as a risk factor for dropout of psychotherapy (Baruch et al., 1998).

To improve psychotherapeutic care for adolescents with CD and address the abovementioned challenges, our workgroup adapted Mentalization-based therapy for application with adolescents with CD (MBT-CD, Taubner and Hauschild, 2021). Mentalization-based treatment (Bateman and Fonagy, 2010) was originally developed with specific vulnerabilities of individuals with Borderline Personality Disorder (BPD) in mind, and aims at enhancing the patients’ mentalizing, i.e., their capacity to understand themselves and others on the basis of mental states. MBT-CD aims at specifically reducing vulnerabilities in the mentalizing of adolescents with CD. Moreover, by using the not-knowing and empathic MBT stance and interventions which are not focused on controlling behavior, an increase in patients’ agency and interpersonal trust is targeted. In a feasibility study, we investigated the acceptability of MBT-CD by the participating adolescents (Hauschild et al., 2023): 43% of adolescents who started MBT-CD dropped out. While the clinical goal of a lower dropout-rate is evident, this rate lies well within the range of dropout-rates identified for outpatient psychotherapy with adolescents (de Haan et al., 2013; O’Keeffe et al., 2018; Jørgensen et al., 2021). Moreover, not to be neglected, 57% of adolescents with CD completed the treatment. Thus, it seems of great value to shed some light on characteristics of adolescents with CD who dropped out of treatment. Yet, it seems just as interesting to raise the question what characteristics may have contributed to more than half of the adolescents staying in therapy, despite the fact that the sample mostly “checked the boxes” of identified risk factors for therapy dropout (such as being adolescent, displaying CD symptom behavior, and most not having come to therapy upon their own initiation). However, to the best of our knowledge, research on potential characteristics of individuals with CD who dropout and/or stay in treatment is lacking (cmp. Bakker et al., 2017). In group-based MBT for adolescent patients with BPD, low mentalizing in patients was predictive for dropout of treatment (Jørgensen et al., 2021). For patients with BPD specifically and psychotherapy patients more generally, a good therapeutic alliance, especially early in treatment, as well as an affective communication style seem to be vital for patients completing therapy; and a lack thereof seems to be an indicator for dropout (Sharf et al., 2010; Barnicot et al., 2011; de Haan et al., 2013). More specifically, qualitative analysis of treatment evaluation interviews with MBT-CD completers in our feasibility study provided some insight into positive and negative aspects of treatment from the patients’ point of view (published only in German language: Hauschild et al., 2021; Hauschild et al., 2023): feeling understood in the therapeutic relationship was amongst the positive aspects. As negative some patients pointed out that they found the questions annoying, and younger patients liked the more structured treatment phase in the beginning (incl. Psychoeducation with their family) better than the less structured individual sessions. Thus, at this point in treatment and research on adolescents with CD, it seems of value to gain a deeper understanding of factors possibly fostering treatment success.

While several aspects point to adolescents with CD having high risk of dropout of therapy, so far, little is known about specific characteristics of adolescents within the CD group which may play a role in these adolescents engaging in therapy or not. Considering the status quo of research on this topic as well as the small sample size, this study aims to explore baseline characteristics CD such as symptom severity and personality pathology of adolescent patients with CD referred to MBT-CD and the occurrence of dropout rates in different “types” of adolescents. As outlined above, previous studies have hinted at these variables influencing dropout behavior. Moreover, in an exploratory stepwise logistic regression, potential predictors of dropout within this patient group are explored.

## Materials and methods

### Participants

Adolescents with a CD diagnosis (F91 group according to ICD-10, including oppositional defiant disorder (ODD), which according to the DSM represents a milder CD version) were included into the feasibility study (Hauschild et al., 2023). Patients were excluded from participation when they had committed sexual offenses, showed acute psychotic symptoms, early-onset schizophrenia, neurological or intelligence impairments, were non-German-speaking or when other clinical contraindications for outpatient psychotherapy existed (e.g., acute suicidality). Of *N* = 32 out of 42 adolescents participating in the feasibility study, baseline questionnaire data were available and used for this study. Of those 32, *n* = 11 (34%) were dropouts, *n* = 21 (66%) were therapy completers. 22 were male and 10 female. *N* = 27 fulfilled criteria of CD, *n* = 5 fulfilled criteria of ODD according to the DSM. Mean age was 14.0 (SD = 1.9, range: 11–18 years). *n* = 4 did not go to school. For *n* = 3, differentiation between school types was not possible. *n* = 3 went to special school, *n* = 10 to lower secondary school, *n* = 5 to higher secondary school, and *n* = 7 to high school. For all but one subject, adolescents were referred to therapy by third parties (e.g., social services, youth homes, psychiatric institutions).

### Procedures

This study is conducted as a secondary analysis of data collected in the feasibility study on MBT-CD (Hauschild et al., 2023), which was approved by the Ethics Committee of the Medical Faculty of Heidelberg University (Germany) (Ethics approval number: S-534/2016) and registered at clinicaltrials.gov (NCT02988453). The feasibility study was conducted from September 2016 to December 2021 at two study sites in Germany: At the Institute for Psychosocial Prevention and Psychotherapy of the University Hospital Heidelberg, Centre for Psychosomatic Medicine, and at the Rheinhessen Fachklinik, Department of Child and Adolescent Psychiatry and Psychotherapy of the University Medical Centre Johannes Gutenberg University, Mainz. Diagnostic assessments were carried out by a psychologist at the beginning and end of treatment and included the CD and ODD sections of the mini-international neuropsychiatric interview for children and adolescents (MINI-KID) (Sheehan et al., 2010) and Semi-structured Clinical Interview of the Diagnostic and Statistical Manual of Mental Disorders (American Psychiatric Association, 2013). Moreover, participants were asked to fill out questionnaires at the beginning, during and at the end of treatment as well as 3 months after the end of treatment. Adolescents received a total of 50€ for taking part in the scientific assessments. Both adolescents and their parents gave written informed consent before participating in the feasibility study. For this study, only the questionnaire data from the beginning of treatment were used.

### Intervention

Adolescents received MBT-CD in the feasibility study, which comprised one individual session per week and one family (or other caretakers) session per month. Flexibility in duration from 6 to 12 months of treatment was exhibited to provide tailored care for each patient. Two psychoeducational sessions for the adolescent and their family were conducted at the beginning of MBT-CD, which included the topics of mentalizing and reciprocal effects of difficulties with mentalizing and dealing with emotionally challenging situations. Building on the information delivered in the psychoeducational sessions, recovery or establishment of mentalizing in such situations was subsequently and consistently targeted in the individual and family sessions. For this purpose, the adolescents’ mentalizing resources and difficulties were written down in a case formulation in form of a letter to the patient in the first sessions after the psychoeducation. Together with the adolescent, the case formulation was worked through, changed where appropriate and agreed upon as therapy focus. Throughout the treatment, therapists worked collaboratively with youth welfare services, if these were involved. Therapists at the study site in Heidelberg (*N* = 8, seven female) were trained in psychodynamic therapy and had a mean age of 35.1 (SD = 8.1). Therapists at the study site in Mainz (*N* = 6, all female) were trained in cognitive behavior therapy (*N* = 4) and psychodynamic therapy (*N* = 2) and had a mean age of 31.7 years (SD = 4.8). All therapists received an MBT-CD training prior to therapy start and received biweekly to monthly supervision, both provided by the last author (ST) and supported by the first author (SH).

### Measures

The Subtypes of Antisocial Behavior questionnaire (STAB), Level of Personality Functioning Questionnaire (LoPF-Q), Reactive-Proactive Aggression Questionnaire (RPQ), and Youth Psychopathic Traits Inventory (YPI), were used to collect the relevant psychological data. All are self-report scales.

The STAB is a useful instrument developed by Burt and Donnellan (2009) to assess different aspects of antisocial behavior. It comprises three scales, measuring physical aggression, rule-breaking behavior, and social aggression (Burt and Donnellan, 2009). The physical aggression scale consists of 10 items (i.e., “Do you feel like hitting someone right now?”), while the rulebreaking and social aggression scales contain 11 items each (i.e., “Are you gossiping and/or complaining about someone right now?”) (Burt and Donnellan, 2009). Every item is to be rated on a Likert scale with the scores ranging from 1 (never) to 4 (always). A high score indicates a frequent exhibition of antisocial behavior. We used all three scales of the STAB. Cronbach’s 𝛼 of the physical aggression scale were 0.85, rulebreaking also 0.85, and social aggression 0.81.

The RPQ aims at capturing quantitatively the distinction between proactive and reactive aggression in order to gain a fuller picture of the behavioral symptoms in adolescents with corresponding impairments or problems (Raine et al., 2006). It contains 23 items (i.e., “Had fights with others to show who was on top”), each of which is to be rated on a 3-point ordinal scale ranging from 0 (never) to 2 (often). The scores provide an overview of the reactive, and the proactive components of aggression, as well as the total aggression. The higher the score, the higher the level of aggression. Only the RPQ total score was included. In this study, the Cronbach’s 𝛼 of the scale was 0.83.

The LoPF-Q was developed to explore levels of personality functioning, specifically in adolescents (Goth et al., 2018). The underlying approach is dimensional as opposed to a more categorical understanding of personality disorders. It assesses four domains, namely identity, self-direction, empathy, and intimacy, here the first two represent self-related functioning and the last two interpersonal functioning. The LoPF-Q consist of 97 items that are to be rated using a Likert scale ranging from 0 (no) to 4 (yes). Statements for the evaluation of the identity functioning were “I am confused about the person that I really am” (item 89), while the functioning on the self-direction subscale were assessed with assertions such as “I have difficulties to reach the goals that I set for myself” (item 24). Patients with a lack of empathy may have agreed with statement such as “I often do not understand other people’s reaction to my behavior” (item 25), and may feel like “I prefer others not to get too close to me” (item 33), if they have an impairment in intimacy. A high score on the LoPF-Q on one of the primary scales indicates a high level of impairment regarding that area of personality functioning. All four primary scales were used in our study. Cronbach’s 𝛼 of the identity scale was 0.85; Cronbach’s 𝛼 of the self-direction scale was 0.91; Cronbach’s 𝛼 of the empathy scale was 0.81; and Cronbach’s 𝛼 of the intimacy scale was 0.78.

The YPI assesses psychopathic traits on 10 subscales, grouped into 3 dimensions: the Callous-Unemotional, the Grandiose-Manipulative, and the Impulsive-irresponsible dimensions (Andershed et al., 2002). Since each subscale contains 5 items, the YPI contains a total number of 50 items, all of which are scored on a 4-point Likert scale, ranging from 1 (Does not apply at all) to 4 (Applies very well). A higher score indicates a higher degree of psychopathy. For our study, we only considered the YPI total scale, with Cronbach’s 𝛼=0.91.

The capacity to mentalize, also referred to as reflective functioning, is the ability to recognize and understand one’s own behavior and the behavior of others as a result of underlying mental states (Fonagy et al., 2002). Mentalizing uncertainty assessed with the RFQ was found to be indicative of clinically impaired mentalizing (Fonagy et al., 2016). In this study, we used the 8-Item version of the RFQ to assess mentalizing uncertainty. To consider economy in scientific assessments, the adult version was chosen as there was to the best of our knowledge no short version of the RFQ for youth at the beginning of the study. Moreover, the wording between adult and youth versions was deemed quite similar (exact same wording in 6 out of 8 items and only small changes in the other 2). However, as internal consistency of the scale was bad with a Cronbach’s 𝛼 of 0.47 in our study, we excluded it from further analyses.

### Statistical analysis

All statistical analyses were calculated with R (version 1.4.1103).

**Figure 1:**
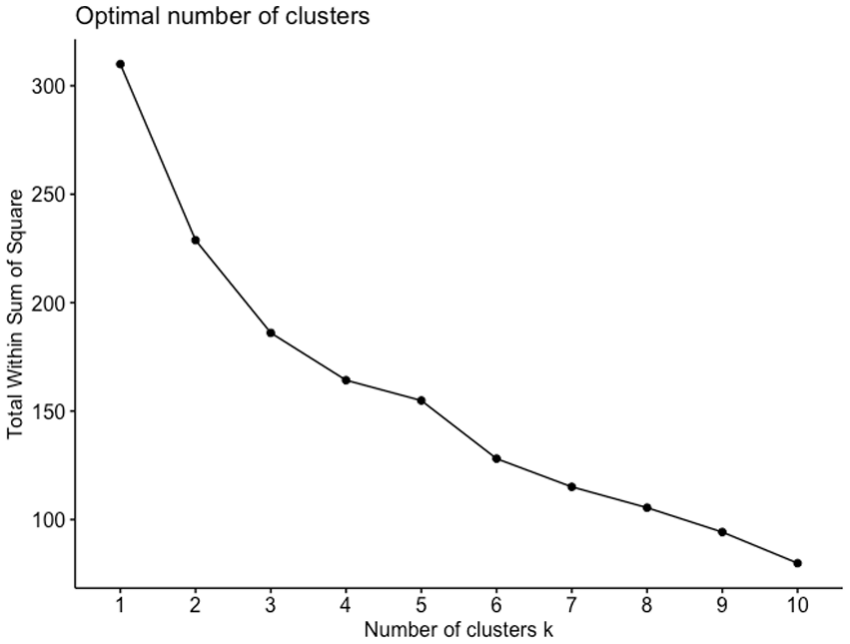
Elbow plot for visualization of numbers of clusters in relation to intra-cluster variation.

We used cluster analysis to explore whether descriptively, there were subgroups in our sample based on the interval scaled participant data (questionnaire data on CD symptom severity, personality pathology, psychopathy and age at the beginning of treatment), and to investigate whether these subgroups show differences in these patient characteristics and come along with different drop-out rates. Cluster analysis represents a partitioning method used to identify meaningful groupings based on a set of specified variables within a dataset (Lund and Ma, 2021). The use of cluster analysis in small samples is controversial; yet, its use for exploring relevant but sparse data is underscored by many in the field of psychological and social sciences (e.g., Henry et al., 2015). Because of the small sample size in this study, the exploratory and descriptive nature of the analysis is the basis of the interpretation of findings. Patterns described here can only be used for hypothesis building and the instigation of future research. The package “factoextra” (version 1.0.7) for R was employed. To determine the number of clusters we first used the Elbow heuristic according to which the appropriate number of clusters is to be determined by the location where the curve bends in the shape of an elbow (Elbow rule). We identified an N of 3 clusters to reach optimal explanation of data variation as the changes in intraclass variation were steeper up to a cluster number of 3 than when adding a 4th or 5th cluster. Also, we chose the cluster number of 3 to avoid overfitting of our data (Figure 1). Then, we ran a 3-means hierarchical cluster analysis using the Ward method and keeping the squared Euclidian distance on the 9 scales assessed via the abovementioned questionnaires as well as the participants’ age as 10^th^ variable. All variables were standardized before clustering.

In a second step, to identify potential predictors for the dichotomous variable dropout, we calculated a stepwise logistic regression (“stats” R package, version 4.04) with 11 candidate variables as predictors (all questionnaire scales used in the cluster analysis plus participants’ age and gender). Type of school was not included into the analysis due to the categorical nature of the variable, which was not dummy-coded. The method of a combination of both, forward and backward selection, was used to find the most contributive predictors. We then calculated Odds Ratios (OR) for significant predictors. The R algorithm uses the Akaike Information Criterion (AIC) determining the amount of information lost in the model, with lower AIC indicating better model fit. To determine goodness of fit we also calculated the Cox and Snell *R*^2^ and Nagelkerke *R*^2^ (standardized Cox and Snell *R*^2^) as “pseudo R^2^s” for logistic regression in analogy to *R*^2^ for linear regression.

## Results

### Cluster analysis

**Figure 2:**
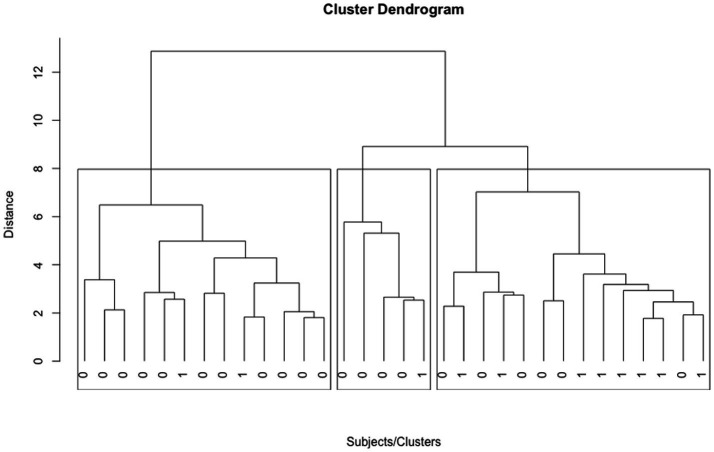
32 subjects in 3 clusters as identified in the cluster analysis. 0 indicates a subject, who completed treatment. 1 indicates a subject, who dropped out of treatment. Cluster 3 is depicted left, Cluster 2 in the middle, Cluster 1 on the right.

Of the 32 participants *n* = 14 were assigned to Cluster 1, *n* = 5 to Cluster 2, and *n* = 13 to Cluster 3 (see Figure 2).

In Table 1 we present sociodemographic characteristics in the three clusters.

**Table 1:** Sociodemographic characteristics across the 3 Clusters.

	Cluster 1 (*n* = 14)	Cluster 2 (*n* = 5)	Cluster 3 (*n* = 13)
CD / ODD diagnosis	12 / 2	5 / -	11 / 2
Heidelberg / Mainz	8 / 6	3 / 2	7 / 6
Dropout	8 (57.1%)	1 (20.0%)	2 (15.4%)
Age (years)	13.9 (2.0)	15.4 (1.8)	13.6 (1.7)
**Gender**			
Male	8 (57.1%)	5 (100%)	9 (69.2%)
Female	6 (42.9%)	0 (0.0%)	4 (30.8%)
**Current education**			
Lower secondary (Hauptschule, Werkschule)	4 (28.6%)	1 (20.0%)	5 (38.5%)
Higher secondary (Realschule)	1 (7.1%)	1 (20.0%)	3 (23.1%)
High school (Gymnasium)	2 (14.3%)	2 (40.0%)	3 (23.1%)
Special needs school	3 (21.4%)	0 (0.0%)	0 (0.0%)
Differentiation not possible	2 (14.3%)	0 (0.0%)	1 (7.7%)
No school	2 (14.3%)	1 (20.0%)	1 (7.7%)

Values depict mean (standard deviation) for “age,” and *n* (% of participants within cluster) for the other variables. Heidelberg/Mainz refers to the study site where individuals took part.

With *n* = 14 patients, Cluster 1 descriptively was the biggest of the three clusters. Of those, *n* = 8 later dropped out of therapy. This represents a drop-out rate of 57.1%, descriptively revealing by far the highest drop-out rate among the three clusters. In addition to the highest dropout-rate, Cluster 1 was descriptively characterized by the strongest personality pathology in the intimacy and self-direction domain. For all clusters, mean scores and standard deviations on the psychometric scales can be found in Table 2.

**Table 2:** Characteristics of the different psychological scales in the three clusters.

	Cluster 1 (*n* = 14)	Cluster 2 (*n* = 5)	Cluster 3 (*n* = 13)
Physical aggression^a^	25.9 (6.7)	38.6 (5.0)	26.0 (7.9)
Social aggression^a^	22.7 (6.8)	27.4 (8.4)	21.5 (6.0)
Rulebreaking^a^	15.9 (4.9)	27.8 (9.0)	17.4 (5.6)
Reactive-proactive aggression^b^	15.3 (4.8)	25.0 (3.8)	10.9 (4.3)
Identity pathology^c^	44.6 (11.7)	45.8 (14.1)	23.2 (11.2)
Self-direction pathology^c^	52.0 (12.5)	40.0 (14.8)	20.3 (10.2)
Empathy pathology^c^	46.4 (6.4)	46.2 (15.4)	26.2 (10.0)
Intimacy pathology^c^	39.1 (8.6)	31.6 (8.1)	19.0 (8.3)
Psychopathy^d^	11.6 (1.5)	14.1 (2.6)	10.5 (1.8)

^aMeasured with the STAB; bmeasured with the RPQ; cmeasured with the LoPF; dmeasured with the YPI.^

Cluster 2 consisted of *n* = 5 participants, rendering it the smallest of the three clusters. *n* = 1 out of those 5 participants, i.e., 20.0%, dropped out of therapy. With this, the dropout rate of Cluster 2 descriptively lies between clusters 1 and 3; yet, Cluster 2 had by far the highest scores on all aggression scales and the psychopathy scale. Thus, Cluster 2 can be considered the cluster with the highest “typical” CD symptomatology. Empathy and identity personality pathology was comparable between Clusters 2 and 1, while pathology in the intimacy and self-direction domain was lower in Cluster 2.

Cluster 3, consisted of *n* = 13 participants. With this, Cluster 3 is the second largest of the three clusters. Only *n* = 2 participants in Cluster 3 dropped out of therapy; i.e., Cluster 3 descriptively showed the lowest drop-out-rate with 15.4%. Cluster 3 was surpassed by Cluster 1 and Cluster 2 in all personality pathology domains, as well as reactive and proactive aggression. Yet, social aggression, rule breaking, and physical aggression as measured with the STAB as well as psychopathy were descriptively comparable between Clusters 3 and 1, indicating similarity in CD symptoms between the two clusters with the highest and lowest dropout rate (57.1% vs. 15.4%).

### Stepwise logistic regression

As shown in Table 3, only one potential predictor for dropout was revealed in the stepwise logistic regression: intimacy personality dysfunction (*ß* = 0.01, *p* = 0.013) came along with an Odds ratio of 1.11 [1.03; 1.22], indicating an 11% increase of the likelihood to dropout with each increase of 1 on the intimacy dysfunction scale. The model (null deviance of 41.183 on 31 degrees of freedom) with intimacy personality dysfunction as predictor had residual deviance of 32.840 on 30 degrees of freedom and an AIC of 36.84. With this, it had a better model fit than the null model (AIC: 43.183), and Nagelkerke *R*^2^ of 0.32 (Cox and Snell *R*^2^: 0.23) indicated a small effect.

**Table 3:** Test model coefficients of stepwise logistic regression calculated to find potential dropout predictors.

	*ß*	SE	Z	*p*	OR [95% CI]	R^2^
Intercept	−3.9	1.5	−2.7	0.007	0.02 [0.00; 0.24]	0.23^a^, 0.32^b^
Intimacy pathology	0.1	0.0	2.5	0.013	1.11 [1.03; 1.22]	

^aCox and Snell; bNagelkerke.^

## Discussion

This exploratory study aimed to investigate baseline characteristics (aggression, personality pathology, psychopathy and age) in adolescents with CD, and their potential relation to treatment dropout. Both, the results of the cluster analysis as well as those of the stepwise logistic regression conducted in this study, hint at low personality functioning, especially in the intimacy domain, as a potentially crucial variable for treatment dropout of adolescents with CD. Aggressive symptoms were not specifically indicative for the cluster associated with the highest dropout rate, nor were they identified as potential predictor of dropout in the stepwise logistic regression. While findings are limited by the small sample size and exploratory character of the analyses, they may be able to inspire future research and provide grounds for shedding increasing light on conducting (successful) psychotherapy with this scarcely researched patient group.

In this study, three clusters were identified in a group of adolescents with CD based on age, self-reported aggression, psychopathy and personality pathology. The clusters came along with different dropout occurrence rates: Cluster 1 comprising 14 adolescents had by far the highest dropout-rate with 57.1%, as compared to the other Clusters with dropout rates of 15.4% (Cluster 2 comprising five adolescents) and 20% (Cluster 3 comprising 13 adolescents). In addition to the highest dropout rate, Cluster 1 descriptively had the highest personality pathology in the intimacy and self-direction domain. Intimacy dysfunction was also revealed as the only potential predictor in the stepwise logistic regression analysis.

Intimacy personality functioning encompasses the capacity to develop close and mutual relationships (Goth et al., 2018). It seems likely, that intimacy dysfunction may have led patients to adopt a distrusting, withdrawn position toward their therapists. Thus, not engaging in a meaningful and potentially long-lasting relationship as it constitutes in therapy may have been a direct result from the symptom itself. Additionally, self-direction pathology, i.e., lack of self-reflection, lack of ability to steer impulses and lack of agency, was highest in Cluster 1. Here as well, it seems likely that such a dysfunction is relevant for engaging in therapy, as therapy often represents a long-lasting, demanding process characterized by emotional ups and downs. Thus, both intimacy and self-direction personality dysfunction are likely to—per definition—represent an obstacle to treatment completion in individuals with CD and need to be taken into account when the treatment with the individual is planned.

It is possible that in some cases, in Cluster 1, patients may have been able to find a way to benefit from the treatment, as some adolescents in this cluster with the highest intimacy and self-direction personality pathology completed the treatment. Potentially, MBT-CD was able to help with specific regard to impairments in the intimacy and self-direction domain. Qualitative data from post-treatment evaluation interviews conducted in the feasibility study (Hauschild et al., 2023) hinted at potential benefits of a positive relationship and more self-direction: when openly asked about helpful and disturbing aspects of their MBT-CD treatment, some adolescents mentioned as positive that they felt understood by their therapist and gained more self-control via an increased ability to reflect on themselves and others. As MBT-CD was developed with vulnerabilities presenting as avoidant attachment and lack of agency in mind (Taubner and Hauschild, 2021), one may hypothesize that the specific MBT-CD stance, which focuses on non-patronizing and not-knowing in the face of symptom behavior which often elicits patronizing, rule-enforcing behavior from others, may have helped in engaging some of these adolescents into therapy along with interventions tailored to optimally regulate emotional arousal associated with different attachment strategies. This would be in line with the treatment’s aim of enhancing adolescents’ curiosity toward their own minds and establishing agency and interpersonal trust. However, at this point, these notions remain speculative. It should be noted that for the bigger part of adolescents in Cluster 1 described by high intimacy and self-direction dysfunction, therapists were seemingly not able to “get a foot in the door.”

Surprisingly, our findings hint at symptom severity playing a subordinate role in regards to therapy completion or dropout. While Cluster 3 with the lowest dropout rate (15.4%) of the three clusters had notably the lowest personality pathology and reactive-proactive aggression, it showed comparable physical, social, and rulebreaking aggression to Cluster 1. Moreover, psychopathy in Cluster 3 was descriptively only a little lower than in Cluster 1. Thus, there was similarity in CD symptom presentation of physical, social and rulebreaking aggression and psychopathy in the two clusters which came along with the highest and lowest dropout rates. Cluster 2 in contrast might be considered the cluster with the most prototypical CD symptomatology as it was characterized by descriptively the highest average scores on all aggression scales as well as on the psychopathy scale. Yet, dropout was lower than in Cluster 1. More specifically, the dropout rate of 20% in Cluster 2 can be considered normal to even low for an adolescent sample (cmp. O’Keeffe et al., 2018). While empathy and identity personality pathology were similar to that in Cluster 1, intimacy and self-direction functioning was descriptively better in Cluster 2 than in Cluster 1, underscoring the potential hindering influence of specifically intimacy and self-direction pathology in individuals with CD for benefitting from psychotherapy. However, it seems important to emphasize again that speculations must be done with caution, as Cluster 2 comprised only 5 individuals.

Taken together, our findings provide grounds for establishment of the hypothesis, that treatment dropout or completion may not as much be influenced by severity of aggressive behavior as it may be by personality pathology, especially in the intimacy domain. With reference to the progredient course of CD, CD has been compared with personality pathology (Fairchild et al., 2019). Taking into consideration the differences in personality pathology that were descriptively revealed in the three clusters in this study, it seems reasonable to assume, that in some cases, CD symptoms may represent an emerging personality pathology. With some individuals with emerging personality pathology, it may be more difficult to establish a stable therapeutic relationship, especially when the dysfunction is pronounced in the intimacy domain, and/or the therapist does not have the means to adapt their interventions accordingly. Adapting interventions to adolescents with CD is highly relevant, as has been shown in a systematic case study (Kasper et al., 2024).

To aid therapists in intervening adequately and providing an optimal atmosphere for their patients with CD, it seems fruitful to conduct thorough, dimensional personality diagnostic with adolescents who show aggressive behavior. Odds ratios of the stepwise regression pointed to high relevance of small differences in intimacy dysfunction for dropout: with each increase of 1 on the intimacy dysfunction scale—which is quite fine-grained and had a range of 9 to 50 in this study—, there was an 11% increase in the likelihood of dropping out of treatment. Also, adolescents with high identity pathology may even be quite inclined to building a therapeutic relationship and experience it as helpful as they might be able to benefit from being supported by an adult in the forming of their identity.

Beyond these findings, it seems plausible that other characteristics of the treatment as well as some patient characteristics have also potentially fostered or impeded treatment success: First, the age range in the study covered younger and older adolescents with very different maturity levels. While the MBT-CD manual implemented in the study flexibly allowed certain adaptations according to the adolescents’ age and maturity, adolescents statements in the post-treatment interviews also indicated that the relatively unstructured phases especially in the middle and end of treatment may have worked better with older adolescents instead of younger ones (Hauschild et al., 2023). In line with this hypothesis, Cluster 1 with the highest dropout rate was on average descriptively younger than Cluster 2, which was also characterized by high personality pathology but a lower dropout rate. Moreover, most therapists conducting MBT-CD in the feasibility study were new to the specific MBT-CD approach. This may be of relevance as obstacles in the training of MBT have been pointed out (Sharp et al., 2020). Thus, insufficient practice in applying the MBT stance and interventions may have in some cases contributed to difficulties in establishing a therapeutic relationship with adolescents as aimed for in MBT-CD.

While our study primarily focused on patient characteristics and their potential relevance to dropout of psychotherapy, our findings can also be discussed within the broader context of the role of therapeutic alliance in dropout of psychotherapy. Specifically, intimacy function or dysfunction, as an individual trait with strong interpersonal implications, may hinder the formation of a strong therapeutic alliance in the treatment of individuals with conduct disorder (CD), thereby complicating the therapeutic process. This is in line with findings in the broader realm of psychotherapy research, where poor quality of the therapeutic alliance has been identified as one of the most stable predictors of dropout of psychotherapy (e.g., Sharf et al., 2010). Interestingly, 80% of the observed dropout in the feasibility study occurred prior to the third individual session of the adolescents’ therapy. Thus, adolescents’ acceptability of MBT-CD might have depended strongly on the very first contacts with the therapists. This may underscore the salient role of the therapeutic alliance in the treatment of these individuals from the very beginning.

Furthermore, within the broader framework of mentalizing theory, the concept of “epistemic trust,” defined as the trust in information communicated by another person (Fonagy and Allison, 2014), may be closely related to intimacy function, potentially influencing patients’ ability to engage in and benefit from a psychotherapeutic relationship. Individuals with CD may have low epistemic trust in information provided by others, which may lead to a biased (hostile) interpretation of the other’s intent in an interpersonal exchange (Talia et al., 2021). On the basis of low epistemic trust, it may only be reasonable to be highly suspicious of or withdraw from a psychotherapeutic relationship. Moreover, individuals with CD may have the (justified) expectation that others have low epistemic trust in them, believing that the information they communicate will not be regarded as trustworthy or relevant. This in turn may cause individuals with CD to communicate in a controlling, aggressive manner, to ensure that the information they convey is deemed relevant – regardless of the other person’s trust or mistrust (Talia et al., 2021). While we did not investigate therapeutic alliance or epistemic trust in this study, placing our findings in this broader realm highlights the importance of future investigation on the dynamics of the relationship between individuals with CD and their therapists, including the role of their mutually shared epistemic trust.

All in all, it seems likely that treatments with individuals with aggressive behavior will benefit from a treatment rationale focused on building a helpful therapeutic alliance tailored to individuals with impairments in personality functioning. This will aid in reducing pessimism and stigma around the psychological treatment of individuals with aggressive behavior. Taken together, the findings of our study suggest that individuals with CD may be clustered into different clinical “types,” which may help better understand individuals’ potential needs and vulnerabilities. It seems necessary to deepen our understanding of aggressive behavior. Severity of aggressive, or CD, symptoms may not represent a barrier to a successful therapy as long as patient and therapist manage to build a strong and meaningful relationship. Treating personality dysfunction possibly underlying aggressive symptom behavior may be crucial in doing so. Herein, interventions might especially focus on improving intimacy and self-direction pathology for adolescents with CD. Hopefully, this will help to reduce treatment pessimism and stigma associated with individuals displaying aggressive symptom behavior as is explicitly promoted by several other workgroups on MBT approaches (Fonagy et al., 2020; Flaaten et al., 2024).

### Limitations

One major limitation of the study is the small sample size. Findings need to be interpreted with caution on the basis of the exploratory nature of the study: They cannot be used to draw conclusions, but only for hypothesis building as they are only descriptive. Patterns identified in the cluster analysis need to be confirmed or denied as meaningful for individuals with CD symptoms in future studies with larger samples. Moreover, while we were amongst other research questions interested in predictors of dropout, our sample included only 11 patients who dropped out of treatment, so that this investigation is called for on a larger sample. However, we believe the potential insights gained from this exploratory analysis in this scarce sample justify our approach, as they can lay the ground for the forming of a deeper understanding of therapeutic success with individuals with CD beyond symptom severity, thereby potentially reducing bias.

Another limitation of the study is the fact that we only used self-report data. As a lot of studies have shown that self-report data diverge from reports of other sources on CD symptoms (e.g., Bakker et al., 2017), future studies should integrate and investigate multiple perspectives. In addition, it would be important to also consider the adolescents’ mentalizing ability as a further factor, as this has already been shown to be important in connection with dropout in other studies (Jørgensen et al., 2021). Unfortunately, due to lack of reliability of the measure in our study, we could not include this potentially relevant variable in our analyses. While the scale has been critizized before for its psychometric properties (Müller et al., 2022), the use of the 8-Item version for adults may have contributed to the bad consistency in our sample of adolescents. Including reliable mentalizing measures should be focused on in future studies on this topic.

## Data Availability

The data analyzed in this study is subject to the following licenses/restrictions: the data are obtained from a vulnerable group who did not give consent for transferral of data to third parties and can therefore not be made available. Requests to access these datasets should be directed to sophie.hauschild@med.uni-heidelberg.de.
